# Analgesic Efficacy of Pre-operative Pregabalin in Dacryocystorhinostomy Surgery: A Systematic Review and Meta-Analysis of Randomized Placebo-Controlled Trials

**DOI:** 10.7759/cureus.48720

**Published:** 2023-11-13

**Authors:** Raghad Alhajaji, Shahad Alshamrani, Sammar Jalal, Amal Habhab, Mohammed A Almahmudi, Hayfaa Alhazami, Roaa Alkanderi, Mubarak M Althaidy, Ahmad Alenezi, Fatma I Al Muhaileej, Abdulatif Abdulrahim

**Affiliations:** 1 Public Health, Makkah Health Affairs, Ministry of Health, Makkah, SAU; 2 Family Medicine, Alhajj Primary Health Care, Ministry of Health, Makkah, SAU; 3 General Dentistry, Umm Al-Qura University, Makkah, SAU; 4 Health Programs Administration, Makkah Health Affairs, Ministry of Health, Makkah, SAU; 5 Medicine and Surgery, Kuwait Institute for Medical Specializations, Kuwait, KWT; 6 General Surgery, Sheikh Jaber Al-Ahmad Al-Sabah Hospital, Kuwait, KWT

**Keywords:** meta-analysis, pain, dacryocystorhinostomy, analgesia, pregabalin

## Abstract

Dacryocystorhinostomy (DCR) is an effective surgical procedure for addressing lacrimal drainage problems. However, it can be a painful operation that involves incisions both inside and outside the eye, often leading to a high incidence of postoperative nausea and vomiting. Preemptive analgesics can be employed to alleviate this unrelieved pain. Nonetheless, many of the drugs used can induce a wide range of adverse effects. Therefore, the aim of this systematic review and meta-analysis is to assess the current evidence regarding the efficacy of pregabalin in managing postoperative pain following DCR surgery. We conducted a thorough search of five electronic databases, namely, PubMed, Web of Science, Scopus, Cochrane, and Google Scholar, to identify relevant randomized controlled trials (RCTs) published before September 2023. The quality of the included studies was assessed using the Cochrane Risk of Bias tool for RCTs. The outcomes we evaluated included postoperative pain, surgery duration, time to first analgesia, total pethidine consumption, and postoperative nausea and vomiting (PONV). Continues data reported as mean difference (MD), and dichotomous data reported as risk ratio (RR), with 95% confidence interval (CI). A pooled meta-analysis of three RCTs, including 240 patients in both the pregabalin and placebo groups, was conducted. The results revealed that the pooled MD in pain scores was significantly lower in patients treated with pregabalin compared to those receiving a placebo ((MD = -1.35 (95% CI: -1.83 to -0.87, p < 0.00001)). Additionally, the pooled MD of pethidine consumption was significantly lower in patients treated with pregabalin compared to those receiving a placebo (MD = -54.13 (95% CI: -103.77 to -4.50, p = 0.03)). However, there was no statistical significance between both groups in terms of time to first analgesia and duration of surgery (p > 0.05). On the other hand, the pooled RR of PONV was significantly lower in patients treated with pregabalin compared to those receiving a placebo (RR = 0.37 (95% CI: 0.24-0.57, p < 0.001)). This meta-analysis demonstrates that pregabalin is an effective and well-tolerated intervention for reducing postoperative pain and PONV following DCR surgery, without significantly affecting surgery duration or time to first analgesia. These findings support the use of pregabalin in improving patient comfort and outcomes in this surgical context.

## Introduction and background

Nasolacrimal duct occlusion causing excessive tearing is a common ophthalmic issue. The preferred treatment for primary or secondary adult anatomical obstruction is dacryocystorhinostomy (DCR), which remains the gold standard for lacrimal surgery. It can be performed under either general or local anesthesia [1-3]. The surgical procedure presents significant challenges and often leads to tissue damage, as well as postoperative nausea and vomiting (PONV). Consequently, it is important to ensure a peri-operative period that is free of stress, accompanied by appropriate pain management, and a minimal occurrence of PONV [4]. Surgery causes tissue injury, resulting in the production of inflammatory mediators that stimulate pain receptors. These stimuli from tissue injury are transmitted by peripheral nociceptors to the spinal cord and higher centers, giving rise to the perception and affective component of pain [5]. After the surgical incision, central sensitization and hyperexcitability may develop, amplifying postoperative pain and potentially leading to adverse postoperative outcomes [6].

Effective analgesic treatment can lead to short-term and long-term improvements in recovery, enhancing the quality of life during a patient's convalescence [7]. Preemptive analgesics are valuable tools for managing unrelieved pain, and they include a range of options, such as local anesthetics, opioids, nonsteroidal anti-inflammatory medications, cyclooxygenase-2 inhibitors, gabapentin, pregabalin, clonidine, and dexmedetomidine [8-10]. Nonsteroidal anti-inflammatory drugs (NSAIDs) are effective in treating mild-to-moderate pain following eye surgeries. However, it is important to note that these medications come with certain adverse side effects, including impaired renal function and the risk of gastrointestinal hemorrhage [11,12]. Opioids continue to be the cornerstone of perioperative pain management. Their careful use is essential for achieving effective analgesia through both central and peripheral mechanisms. Nonetheless, opioids are associated with several side effects, such as PONV, sedation, drowsiness, itching, and delayed recovery, which can increase healthcare costs [13,14].

Pregabalin possesses both anticonvulsant and anxiolytic properties. It is a lipophilic gamma-aminobutyric acid (GABA) analog that binds to voltage-gated calcium channels. This binding reduces calcium entry into central nervous system nerve terminals and lowers inflammatory mediators that contribute to pain perception [15-17]. Pregabalin has demonstrated effectiveness across a range of conditions, including neuropathic pain, incisional injury, and inflammatory injury models, such as tissue irritation, neuralgia, and fibromyalgia [17]. The preoperative administration of pregabalin is seen as a promising strategy to improve postoperative pain control and reduce the need for postoperative opioids. Given its relatively mild side effects such as dizziness, drowsiness, and dry mouth, and its potential for both short-term and long-term benefits, pregabalin emerges as a valuable option for managing mild-to-moderate pain in surgical patients [18,19]. The objective of this systematic review and meta-analysis is to consolidate current evidence regarding the safety and efficacy of pregabalin in managing postoperative pain following DCR surgery.

## Review

Methods

This study has been conducted strictly with the standards outlined in the PRISMA statement [20]. All procedures were implemented in complete adherence to the guidelines outlined in the Cochrane Handbook of Systematic Reviews and Meta-analysis of Interventions (version 5.1.0) [21].

Eligibility Criteria

Our review incorporated studies that met the following inclusion criteria: a) population: studies involving adult patients diagnosed with nasolacrimal duct obstruction undergoing DCR surgery; b) intervention: utilization of pregabalin for pre-operative analgesia, compared to placebo pills; c) outcomes: assessment of the impact of pregabalin on post-operative pain, time to the first request for analgesics, duration of surgery, total pethidine consumption (mg), and the incidence of PONV; and d) study design: we exclusively included randomized controlled trials (RCTs).

We applied the following exclusion criteria: studies involving pediatric patients or individuals with conditions other than nasolacrimal duct obstruction; studies utilizing interventions other than pregabalin for pre-operative analgesia; studies with outcomes not related to post-operative pain, time to the first analgesic request, or PONV; studies with non-randomized designs, observational studies, case reports, or reviews; studies presented solely as abstracts or theses; and studies not published in the English language.

Information Sources and Search Strategy

We conducted a comprehensive search in five electronic databases (PubMed, Scopus, Web of Science, Cochrane Central Register of Controlled Trials, and Google Scholar) from their inception until the 18th of September 2023. The search query, as outlined in Table 1, was used to identify relevant studies. In addition to the electronic database search, we performed a systematic manual search of the references in the included research articles to identify any potentially eligible studies. This approach ensured a thorough exploration of the existing literature. Furthermore, a comprehensive examination of the references in the mentioned studies, along with a thorough review of clinicaltrials.gov, the WHO Clinical Trials registration, and ResearchGate, was conducted to ensure meticulous screening and minimize the risk of overlooking any pertinent publications.

**Table 1:** The precise literature search strategy used in every database.

Databases search strategy
PubMed All Fields: (dacryocystorhinostomy OR “dacryocystorhinostomy surgery” OR dacryocystorhinostom*) AND (pregabalin OR lyrica OR “CI1008” OR “(S+)-3-isobutyl GABA” OR “3-(aminomethyl)-5-methylhexanoic acid” OR “3 isobutyl GABA”).
Scopus Article title, Abstract, Keywords: (dacryocystorhinostomy OR “dacryocystorhinostomy surgery” OR dacryocystorhinostom*) AND (pregabalin OR lyrica OR “CI1008” OR “(S+)-3-isobutyl GABA” OR “3-(aminomethyl)-5-methylhexanoic acid” OR “3 isobutyl GABA”).
Web of Science All Fields: (dacryocystorhinostomy OR “dacryocystorhinostomy surgery” OR dacryocystorhinostom*) AND (pregabalin OR lyrica OR “CI1008” OR “(S+)-3-isobutyl GABA” OR “3-(aminomethyl)-5-methylhexanoic acid” OR “3 isobutyl GABA”).
Cochrane CENTRAL Title Abstract Keyword: (dacryocystorhinostomy OR “dacryocystorhinostomy surgery” OR dacryocystorhinostom*) AND (pregabalin OR lyrica OR “CI1008” OR “(S+)-3-isobutyl GABA” OR “3-(aminomethyl)-5-methylhexanoic acid” OR “3 isobutyl GABA”).
Google Scholar All Fields: (dacryocystorhinostomy OR “dacryocystorhinostomy surgery” OR dacryocystorhinostom*) AND (pregabalin OR lyrica OR “CI1008” OR “(S+)-3-isobutyl GABA” OR “3-(aminomethyl)-5-methylhexanoic acid” OR “3 isobutyl GABA”).

Selection Process

To ensure the quality and relevance of the studies included in this meta-analysis, we employed the following screening process: duplicates were initially identified and removed using EndNote (Clarivate Analytics, Philadelphia, PA). The retrieved references were then subjected to a two-stage screening process. Each stage involved independent assessments by both authors to determine the relevance of the articles. In the first stage, the titles and abstracts of the articles were reviewed to assess their potential suitability for inclusion. In the second stage, the full-text articles were assessed to determine their eligibility for the meta-analysis. The Rayyan website (https://www.rayyan.ai/) [22,23] was utilized to facilitate the selection process, enhancing the efficiency and accuracy of our screening procedures. This rigorous screening process helped ensure the inclusion of high quality and relevant studies in our meta-analysis.

Data Collection Process and Data Items

We utilized a standardized data extraction sheet to systematically collect relevant information from the included studies. The extracted data encompassed various aspects, including study characteristics, population characteristics, quality evaluation domains, and outcome measures. The following outcomes were assessed and extracted from the included studies: post-operative pain, measured using the visual analog scale (VAS), which ranges from 0 to 10 (with 0 representing no pain and 10 indicating the worst intolerable pain); duration of surgery; time to the first administration of analgesia; total pethidine consumption in the first 24 hours; and PONV, which represents the occurrence of nausea and vomiting within the first 24 hours after surgery. This comprehensive data extraction process enabled us to capture key information necessary for our meta-analysis.

Assessing the Risk of Bias in Individual Studies

Two independent reviewers evaluated the risk of bias in the RCTs included in the study using the Cochrane Collaboration's Risk of Bias 2 (ROB-2) tool [21]. Authors classified the risk of bias for each study into one of three categories: "low," "high," or "some concern." Domains assessed encompassed the randomization process, deviations from intended interventions, missing outcome data, outcome measurement, selection of reported results, and other potential sources of bias.

Synthesis Methods

For our meta-analysis, we reported the pooled effect as the mean difference (MD) for continuous outcomes and the risk ratio (RR) for dichotomous outcomes. We used RevMan (version 5.4 for Windows; https://training.cochrane.org/online-learning/core-software/revman) to conduct a DerSimonian-Liard meta-analysis, pooling the RR and MD, along with their respective 95% confidence intervals, for all outcomes. The DerSimonian-Liard meta-analysis technique was employed to calculate the combined effect size for all outcomes. This random-effects model assigns greater weight to smaller studies, acknowledging that the included studies represent a random sample from the population. We opted for this model over the fixed-effects alternative as it accounts for a greater standard error in the pooled estimate, which is particularly valuable when dealing with conflicting or contested findings. Consequently, the effects calculated in our meta-analysis should be viewed as cautious estimates. To assess statistical heterogeneity among the studies, we used the chi-square test (Cochrane Q test). The chi-square statistic, Cochrane Q, was calculated, and heterogeneity was categorized as follows: significant heterogeneity was defined as a chi-square P value below 0.1, and I-squared values below 50% indicated high levels of heterogeneity. However, the assessment of publication bias could not be determined due to the inclusion of fewer than ten studies [24].

Results

Literature Search Results

**Figure 1:**
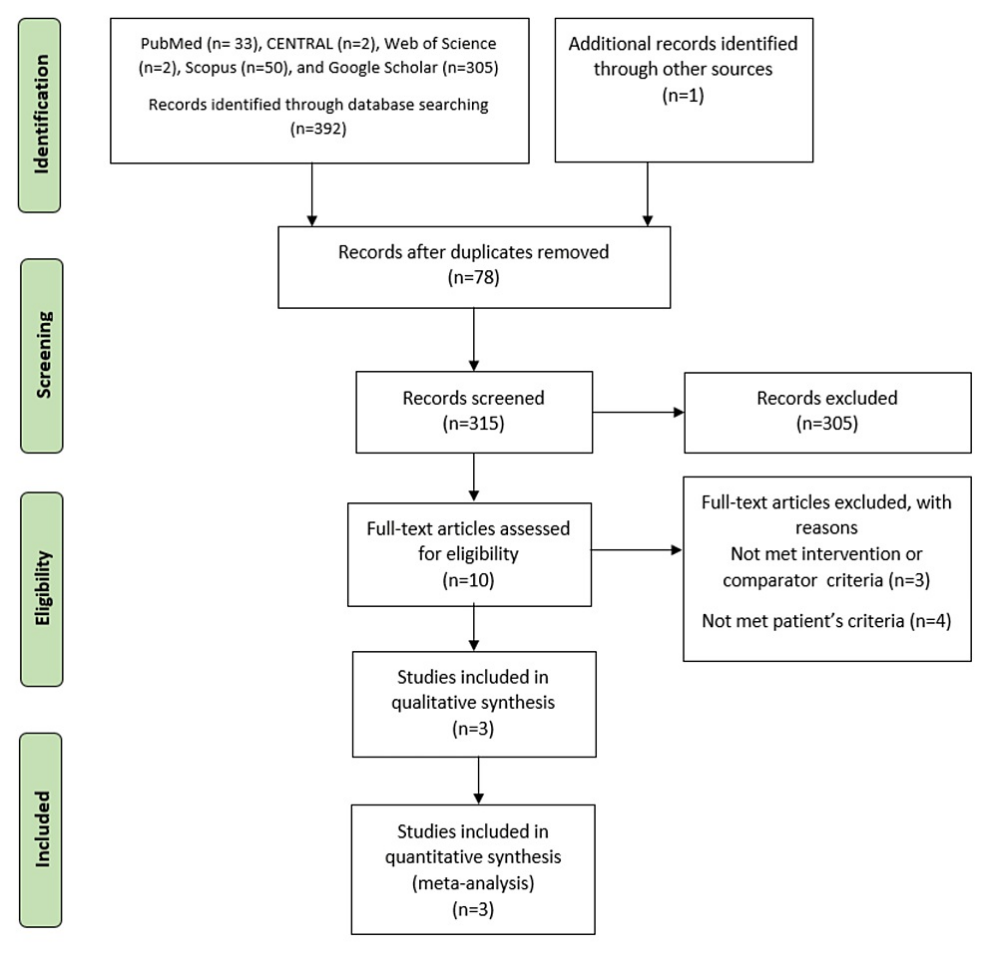
PRISMA flow diagram for the study selection process.

Our literature search process retrieved 315 records. After removing 78 duplicates and conducting title and abstract screening, 305 articles were excluded, leaving 10 articles eligible for full-text screening. Of these, three RCTs [25-27] were included in the meta-analysis. The PRISMA flow diagram illustrating the study selection process is presented in Figure 1.

Characteristics of the Included Studies

Three RCTs were included in the meta-analysis, involving a total of 240 patients who underwent DCR surgery. In all these studies, patients were randomly assigned to receive either pregabalin or a placebo. Two of the RCTs were conducted in Iran, while one was carried out in Egypt. A total of 300 mg of pregabalin was administered in the included RCTs. The assessment of post-operative pain in all these RCTs was done using the VAS score. A summary of the characteristics of the included studies can be found in Table 1 and Table 2.

**Table 2:** Summary of the included studies. Source: Refs. [25-27]

Study	Study design	Country	Sample size	Trial arm		Timing of intervention or placebo
				Intervention	Control	
Alimian et al. 2012 [25]	Double-blind, randomized placebo-controlled trial	Iran	n=80	300 mg of oral pregabalin	Placebo pills	an hour before entering the operation room on the morning of the surgery day
Elsherbiny et al. 2022 [26]	Double-blind, randomized placebo-controlled trial	Egypt	n=100	150 mg of oral pregabalin	Placebo pills	One on the night before the surgery, and the other 2 hours before the surgery
Rimaz et al. 2014 [27]	Double-blind, randomized placebo-controlled trial	Iran	n=60	300 mg of oral pregabalin	Placebo pills	2 hours prior to entering the operating room on the morning of the surgery day

**Table 3:** Baseline information of the included studies. Sources: Refs. [25-27]

Study	Group	Sample size	Age (years)	Sex [male/female]	ASA	Pain assessment tool	Follow-up	
								Alimian et al. 2012 [25]	Pregabalin	n=40	41.1 ± 14.1	[28/12]	[I+II]	Visual analog scale (VAS)	24 hours	
Placebo	n=40	45.4 ± 15.7	[21/19]	[I+II]											
Elsherbiny et al. 2022 [26]	Pregabalin	n=50	43.58 ± 12.18	[20/30]	[I+II]	Visual analog scale (VAS)	24 hours	
	Placebo	n=50	47.16 ± 14.33	[26/24]	[I+II]			
Rimaz et al. 2014 [27]	Pregabalin	n=30	65.56 ± 16.68	[14/16]	[I+II]	Visual analog scale (VAS)	24 hours	
	Placebo	n=30	66.33 ± 15.47	[16/14]	[I+II]			

**Figure 2:**
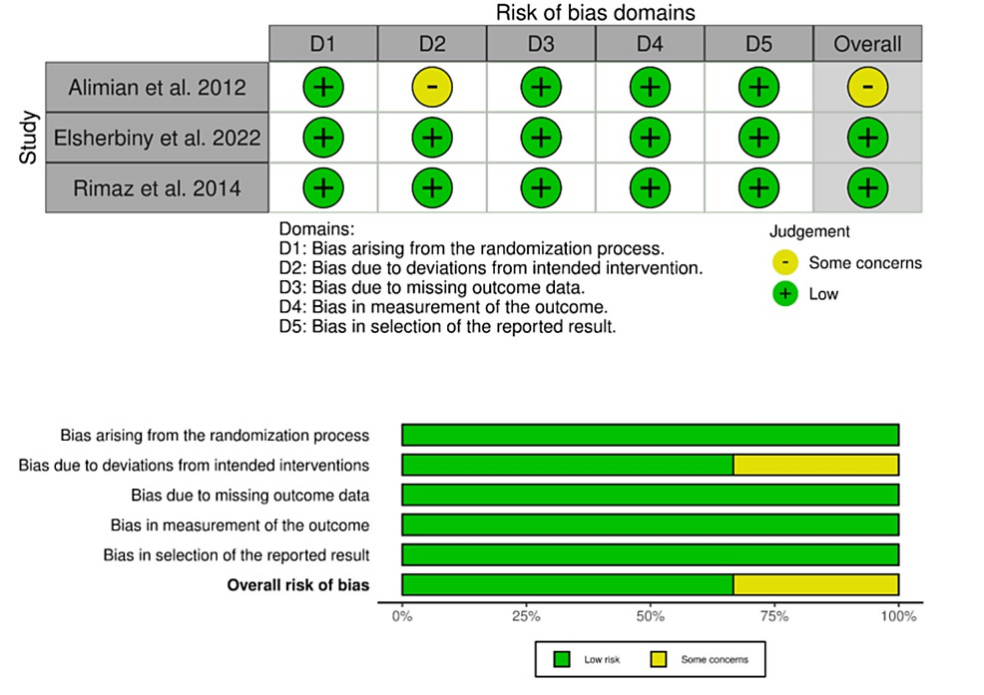
Risk of bias graph and summary of the included studies. Sources: Refs. [25-27]

Overall, the risk of bias in these studies was assessed as low risk (n = 2 RCTs) and some concerns (n = 1 RCTs) according to the Cochrane risk of bias tool, as illustrated in Figure 2.

Outcomes 

**Figure 3:**
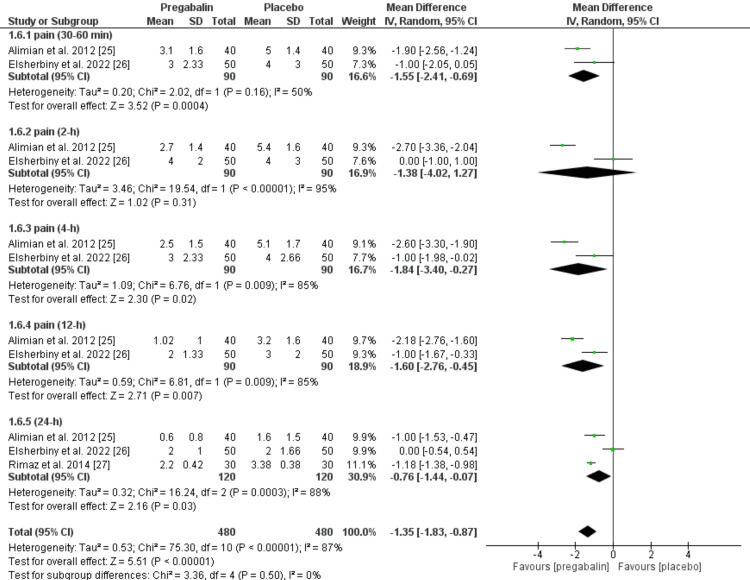
Meta-analysis of the mean postoperative pain (VAS). Sources: Refs. [25-27]

Post-operative pain (VAS): A pooled analysis of 240 patients who received either pregabalin or a placebo revealed a significantly lower MD in pain scores in favor of the pregabalin group compared to the placebo group (MD = -1.35 (95% CI: -1.83 to -0.87, p < 0.00001)). There was also statistically significant heterogeneity. Furthermore, the difference between the two groups was significant, favoring the pregabalin group at various postoperative time points: within 30-60 minutes (MD = -1.55 (95% CI: -2.41 to -0.69, p = 0.0004)), four hours (MD = -1.84 (95% CI: -3.40 to -0.27, p = 0.02)), 12 hours (MD = -1.60 (95% CI: -2.67 to -0.45, p = 0.007)), and 24 hours (MD = -0.76 (95% CI: -1.44 to -0.07, p = 0.03)), as shown in Figure 3. However, there was no significant difference between both groups after two hours (MD = -1.38 (95% CI: -4.02 to 1.27, p = 0.31)).

**Figure 4:**
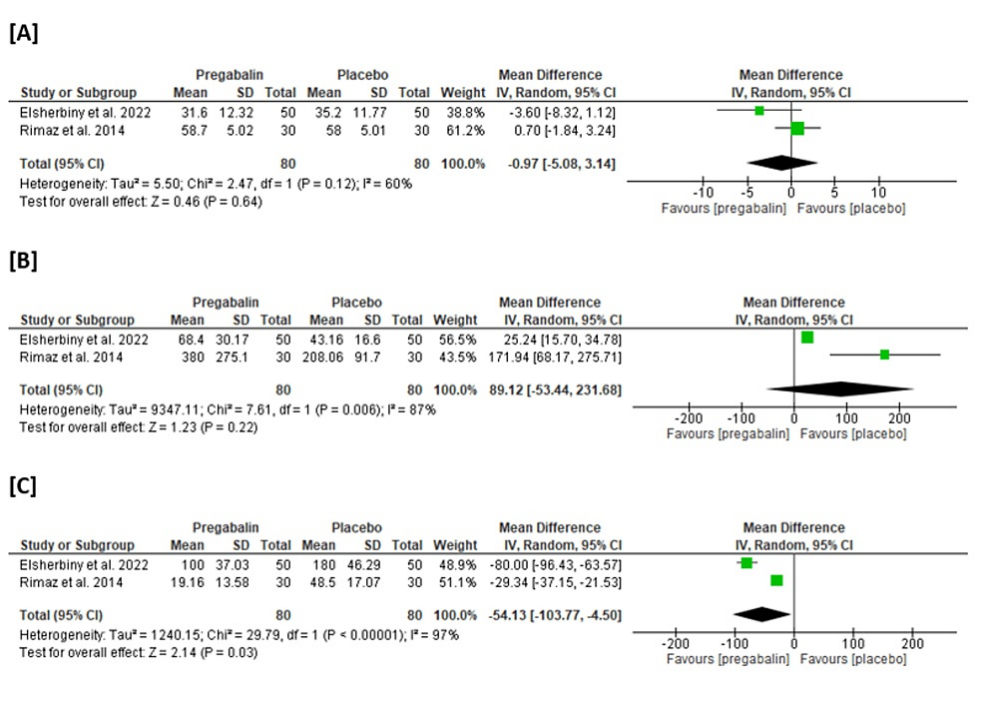
Meta-analysis of the mean change in [A] duration of surgery (minutes), [B] time to first analgesia (hours), and [C] total pethidine consumption (mg). Sources: Refs. [25-27]

Duration of surgery (minutes): Among the 80 patients treated with pregabalin and the other 80 patients treated with a placebo, the pooled MD in the duration of surgery was found to be MD = -0.97 (95% CI: -5.08 to 3.14, p = 0.64), indicating a non-statistically significant change between the pregabalin and placebo groups, and there was no heterogeneity, as illustrated in Figure 4A.

Time to first analgesia (hours): The pooled MD between the 80 patients treated with pregabalin and the other 80 patients treated with a placebo is MD = 89.12 (95% CI: -53.44 to 231.68, p = 0.22), which is non-statistically significant between the two groups, and there is high heterogeneity, as depicted in Figure 4B.

Total pethidine consumption (mg): The pooled MD of total pethidine consumption among 160 patients treated with either pregabalin or placebo showed a statistically significant difference in total pethidine consumption in mg favoring pregabalin over the placebo (MD = -54.13 (95% CI: -103.77 to -4.50, p = 0.03)), and there was statistically significant heterogeneity, as shown in Figure 4C.

**Figure 5:**
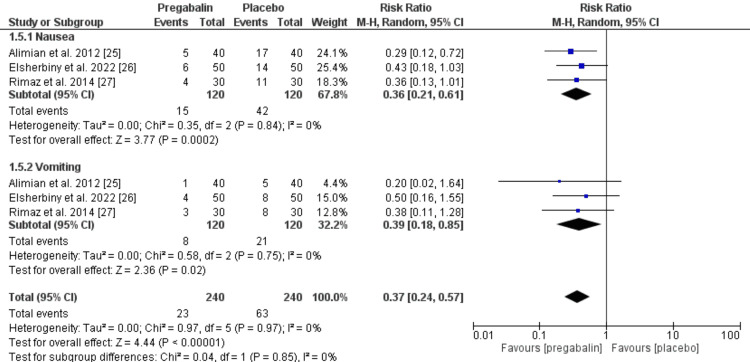
Meta-analysis of the rate of postoperative nausea and vomiting (PONV). Source: Refs. [25-27]

Post-operative nausea and vomiting (PONV): The overall pooled RR of PONV was significantly lower in patients treated with pregabalin compared to those receiving the placebo (RR = 0.37 (95% CI: 0.24 to 0.57, p < 0.001)). On subgroup analysis, 15 patients from the pregabalin group reported nausea, compared to 42 in the placebo group, resulting in RR = 0.36 (95% CI: 0.21 to 0.61, p = 0.0002), with significant heterogeneity among studies. In the case of vomiting, only eight patients in the pregabalin group experienced it, compared to 21 in the placebo group, resulting in a statistically significant RR = 0.38 (95% CI: 0.18 to 0.85, p = 0.02), also with significant heterogeneity among studies, as depicted in Figure 5.

Discussion

This systematic review and meta-analysis included 240 patients who underwent DCR surgery. Our study showed a low risk of bias overall, with two RCTs falling under low risk and one RCT having some concerns. The postoperative pain in the pregabalin group was both clinically and statistically significantly lower compared to the placebo group. This trend was also observed in the total pethidine consumption. However, there was no significant difference between the pregabalin and placebo groups in terms of the duration of surgery and time to first analgesia. In terms of safety outcomes, there was a significant difference favoring the pregabalin group in the rate of PONV.

Uncontrolled postoperative pain can lead to a range of short- and long-term consequences. Optimizing perioperative analgesia not only reduces complications but also supports recovery during the immediate postoperative period and after hospital discharge [28]. Pre-emptive analgesia, administered before an incision is made, has demonstrated greater effectiveness in controlling postoperative pain by protecting the central nervous system from the potentially severe effects of noxious stimuli, allodynia, and hyperalgesia [16]. Recent studies have explored the impact of pregabalin as a pre-emptive analgesic in reducing postoperative pain, mitigating opioid-related side effects, and enhancing patient satisfaction [25,27,29,30]. It is worth noting that the choice of anesthesia and analgesia can vary based on the type of surgery, with the goal of effectively alleviating post-operative pain [31,32]. Pregabalin use has been assessed across different surgical procedures, doses, and timing relative to surgery. However, due to this variability, drawing conclusive recommendations regarding the ideal dosage or duration of treatment from these trials remains challenging. Moreover, the potential long-term benefits of perioperative pregabalin have yet to be thoroughly investigated.

According to the results of this study, pre-operative pregabalin demonstrated superior efficacy compared to a placebo in reducing postoperative pain. Pregabalin exhibited a significant reduction in postoperative pain levels compared to the placebo at 30-60 minutes, four hours, 12 hours, and 24 hours after surgery. Patients who received pregabalin prior to surgery required less pethidine and reported fewer incidents of nausea and vomiting compared to those in the placebo group. However, no statistically significant differences were observed in total surgical time or the time to the first analgesic request when comparing the two groups

The effectiveness of preoperative pregabalin in alleviating postoperative pain can be attributed to its ability to reduce central pain stimulation. Nociceptive receptors are primarily responsible for the natural perception of pain following surgery [16]. However, surgery-induced hyperalgesia can prolong the recovery period after an operation. Pregabalin distinguishes itself from traditional analgesics due to its capacity to attenuate the activation of posterior horn neurons induced by tissue damage [16]. As a result, the preoperative administration of pregabalin is highly recommended to mitigate severe postoperative pain and discomfort. It is important to note that the outcomes of pregabalin analgesia can vary based on factors such as the type of surgery and dosage. In a systematic review conducted by Zhang et al., it was observed that pregabalin doses below 300 mg administered preoperatively did not significantly reduce postoperative pain, while higher doses were effective in reducing pain intensity but also led to an increase in adverse effects [33]. In the case of patients undergoing laparoscopic cholecystectomy, those who received 150 mg of pregabalin 60 minutes before surgery reported significantly less discomfort, as reported by Agrawal et al. [34]. A meta-analysis by Lam et al. included 57 studies with a total of 2,033 patients who received pregabalin and 2,033 patients who served as controls. They assessed the effectiveness of postoperative analgesia and associated risks in various surgical procedures. The studies were divided into two groups based on whether patients received a single dose or multiple doses of pregabalin (beginning the night before or days before procedures). The results indicated that pregabalin, irrespective of the dosage, reduced surgical pain scores for 24 hours, with no significant difference in postoperative pain scores between the two groups [35]. In conclusion, the effect of pregabalin dosage varies depending on the type of surgery, highlighting the need for individualized approaches to pain management.

This finding is consistent with two of the included studies that administered 300 mg of pregabalin prior to DCR surgery [25,27], while only one study used 150 mg [26]; the pooled results favored pregabalin over the placebo [26]. The impact of preoperative pregabalin on postoperative opioid consumption was investigated in a meta-analysis of 46 studies, involving 1,610 pregabalin-treated patients and 1,636 control patients. The analysis revealed that pregabalin reduced total morphine consumption 24 hours after surgery [35]. This finding aligns with the results from Alimian et al., where only seven patients in the pregabalin group required rescue anesthesia compared to 21 patients in the control group [25]. Notably, the likelihood of administering rescue anesthesia in the control group was five times higher than in the pregabalin group among patients undergoing DCR surgery [25]. The lack of a significant effect on the duration of surgery may be attributed to the mechanism of action of pregabalin and other analgesic drugs, which primarily act on the central perception of pain, without affecting the surgical steps and, consequently, the duration of surgery. Similarly, the absence of a significant difference in the time to request analgesia between the two groups may be attributed to the variations in doses and intervals from surgery among the studies, which could introduce heterogeneity and result in non-significant findings.

Regarding the use of pregabalin in the ENT field, Kim et al. conducted a study to investigate its efficacy in septoplasty. They concluded that the perioperative administration of oral pregabalin (150 mg twice) is an effective and safe method for reducing early postoperative pain in patients undergoing septoplasty [36].

To the best of our knowledge, this is the first meta-analysis that compares the effects of pregabalin and placebo on postoperative pain in patients undergoing DCR. However, it is important to note that our study has certain limitations, including a relatively small sample size. As such, we recommend conducting additional trials to more comprehensively assess the evidence and this relationship. Heterogeneity in some of the outcomes may also be a limitation when interpreting the results. Therefore, we encourage further research, particularly studies with more homogeneous surgical dosages and intervals, to provide a clearer and more conclusive understanding of the effects of pregabalin in this context.

## Conclusions

In conclusion, preoperative administration of pregabalin demonstrates a significant reduction in postoperative pain, contributing to improved patient outcomes in DCR procedures. Pregabalin's effectiveness in minimizing pain, reducing opioid consumption, and lowering the incidence of nausea and vomiting underscores its potential as a valuable adjunct in pain management strategies. While our study reveals promising results, further research with larger sample sizes and standardized protocols is warranted to refine the understanding of pregabalin's role in optimizing post-operative care for DCR patients.
